# Chitosan Augments Tetramycin against Soft Rot in Kiwifruit and Enhances Its Improvement for Kiwifruit Growth, Quality and Aroma

**DOI:** 10.3390/biom11091257

**Published:** 2021-08-24

**Authors:** Qiuping Wang, Cheng Zhang, Xiaomao Wu, Youhua Long, Yue Su

**Affiliations:** 1 Research Center for Engineering Technology of Kiwifruit, Institute of Crop Protection, Teaching Experimental Farm, College of Agriculture, Guizhou University, Guiyang 550025, China; qpwang518@aliyun.com (Q.W.); 2111816013@stmail.ujs.edu.cn (C.Z.); xmwu@gzu.edu.cn (X.W.); 2 Department of Food and Medicine, Guizhou Vocational College of Agriculture, Qingzhen 551400, China

**Keywords:** tetramycin, chitosan, kiwifruit, soft rot, adjuvant, yield and quality

## Abstract

In this study, the co–application of chitosan and tetramycin against kiwifruit soft rot and its effects on the disease resistance, growth, quality and aroma of kiwifruit were investigated. The results show that chitosan could effectively enhance tetramycin against soft rot of kiwifruit with the field control efficacy of 85.33% for spraying chitosan 100 time + 0.3% tetramycin AS 5000–time dilution liquid, which was higher than 80.99% for 0.3% tetramycin AS 5000–time dilution liquid and significantly (*p* < 0.01) higher than 40.66% for chitosan 100–time dilution liquid. Chitosan could significantly (*p* < 0.05) improve the promoting effects of tetramycin on total phenolics, total flavonoids, SOD activity of kiwifruit compared to tetramycin during storage for 0–28 days and enhance the disease resistance of kiwifruit. Moreover, the co–application of chitosan and tetramycin was more effective than tetramycin or chitosan alone in enhancing fruit growth, improving fruit quality and increasing fruit aroma. This study highlights that chitosan can be used as an adjuvant to enhance tetramycin against soft rot of kiwifruit and promote tetramycin’s improvement for the single fruit volume and weight, vitamin C, soluble sugar, soluble solid, dry matter, soluble protein, titratable acidity and aroma of kiwifruit.

## 1. Introduction

Kiwifruit (*Actinidia*), as a typical representative of third–generation fruit, is rich in vitamin C and has high nutritional and medicinal values [1]. Recently, the kiwifruit industry has developed rapidly in the world, especially in China, where the planting area and annual output have reached 243,000 hm^2^ and 2.5 million tons, respectively [1,2]. However, postharvest rot diseases caused by pathogens such as *Penicillium expansum*, *Botryosphaeria dothidea* and *Phomopsis* sp. occur frequently because kiwifruit is a typical respiratory climacteric fruit with a thin and juicy skin [3,4]. Soft rot is the most serious disease of postharvest kiwifruit, which is mainly caused by *B. dothidea*, *Phomopsis* sp., *Cryptosporiopsis actinidiae*, *Botrytis cinerea*, *Cylindrocarpon* sp. and *Phoma exigua* [5,6,7,8,9,10,11,12]. In Guizhou Province of Southwest China, kiwifruit soft rot caused by *B. dothidea* and *Phomopsis* sp. seriously affects the quality and yield of kiwifruit and often causes 30–50% economic losses [10,11,12]. Although some chemical fungicides have good antifungal activity and low acute toxicity, their residuals are still potential risks to the environment, wild life and human beings. Moreover, pathogen resistance is easily inducted by chemical fungicides such as thiophanate–methyl, difenoconazole and propineb [13,14]. Therefore, it is urgent and essential to develop green, effective, and economical control strategies of soft rot in kiwifruit.

Tetramycin, the metabolite of *Streptomyces hygrospinosus* var. *Beijingensis*, is a novel medical or agricultural antibiotic containing two active components (tetramycin A and tetramycin B) [15,16]. Owing to its promising antimicrobial activity and eco–friendly advantage, tetramycin was widely used for controlling various plant pathogens in recent years, such as *Botrytis cinerea*, *Colletotrichum scovillei*, *Pyricularia oryzae*, *Phytophthora capsici* and *Passalora fulva* [17,18,19,20,21,22]. In China, tetramycin was registered for controlling fruit and rice diseases, and gradually became a preferred alternative to conventional agricultural antibiotics [12,23,24,25]. Our previous study results indicate that tetramycin exhibited superior toxicity activity against various pathogens in kiwifruit, and possessed good field control efficacies for soft rot, canker, blossom blight and brown spot disease. Meanwhile, it notably increased fruit resistance substance contents, activated fruit resistance–related enzyme activities, as well as effectively enhanced fruit volume and weight, improved fruit vitamin C, soluble sugar, soluble solid, dry matter, soluble protein, titratable acidity and firmness [25]. However, the long–term single use of tetramycin easily leads to the development of pathogen resistance.

Chitosan, the product for removing more than 55% of chitin acetyl groups, is a natural macromolecule compound with antibacterial, nontoxic, antioxidant, renewable and biocompatible advantages [26,27,28]. Many studies have shown that chitosan has excellent biological activity towards various postharvest fruits and vegetable diseases, and was widely used in their storage and fresh–keeping [26,27,28,29,30,31,32]. In our previous study, the chitosan composite film composed of chitosan, dextrin, ferulic acid, calcium and auxiliaries had noticeably preventive and curative effects against kiwifruit soft rot caused by *B. dothidea* and *Phomopsis* sp.; moreover, it increased resistance compounds, activated defense enzyme activity, enhanced yield and quality and prolonged the shelf life of kiwifruit [10,11,12]. Furthermore, chitosan can be used as a resistance inductor and bio–fungicide for inducing a variety of defense–related reactions and controlling plant diseases and as a growth promoter for enhancing plant development [29,30,31,32,33,34]. At present, it is unclear whether tetracycline and chitosan will interact to affect pre– and post–harvest kiwifruit. Thus, it is worth further study that whether chitosan can be used as an adjuvant to enhance tetracycline against soft rot of kiwifruit.

In this study, the control efficacy of the co–application of chitosan and tetramycin on soft rot in kiwifruit was firstly evaluated. Moreover, the effects of the co–application of chitosan and tetramycin on the disease resistance, growth, quality and aroma components of kiwifruit were also investigated. This work provides an effective, green, safe and economical approach for reducing pathogen resistance and controlling kiwifruit soft rot.

## 2. Materials and Methods

### 2.1. Materials

*Botryosphaeria dothidea* and *Phomopsis* sp. with highly pathogenicity were provided by the Research Center for Engineering Technology of Kiwifruit, Guizhou University (Guiyang, Guizhou, China). They were isolated from the *Actinidia delicios*. cv. ‘Guichang’ fruits obtained from the kiwifruit orchard in Xiuwen county, Guizhou province, China, and kept at −20 °C. Chitosan (deacetylation ≥ 90.00%, Huarun Bioengineering Co. Ltd., Zhengzhou, Henan, China), 0.3% tetramycin aqueous solutions (AS) (Liaoning Microke Biological Engineering Co. Ltd., Chaoyang, Liaoning, China). Potato dextrose agar (PDA, g L^–1^): potato (200 g), dextrose (20 g), agar (15 g), distilled water (1 L), pH value was natural, and PDA was sterilized at 121 °C for 30 min.

### 2.2. In Vitro Toxicity Tests

The in vitro toxicity of chitosan and tetramycin for pathogens was determined according to Zhang et al. [11,12]. Furthermore, 9 mL fresh PDA was fed into Petri dishes with 90 mm diameter. After PDA solidification, 1 mL tested solution of tetramycin or chitosan was uniformly coated on the PDA plate by a sterilized glass coating rod, and the control was sterile water. Subsequently, a pathogen disc with a diameter of 5 mm was cut from the active growth front end of a 7–day–old pathogen PDA plate, and it was placed in the new PDA plate center with the inoculum side down and three replicates. The treated plate was then cultured at 28 °C till the fungal growth almost covered the control plates, the diameters of the fungal growth were determined. The growth inhibition of fungal hyphae was calculated as Equation (1):

Inhibition rate (%) = 100 × ((Mycelial growth diameter in control dish − Mycelial growth diameter in treatment dish)/(Mycelial growth diameter in control dish – 5))(1)

EC_50_ (effective concentration of 50% inhibition rate) values were calculated statistically by SPSS 18.0 software.

### 2.3. Field Experiments

#### 2.3.1. Study Site

Field experiments were carried out in 2020 in a kiwifruit orchard with a tree age of 6 years of cultivar *A. delicios*. cv. ‘Guichang’ in Xiuwen country, Guizhou province, China (26°79′80.0″ N, 106°56′58.2″ E). Concrete ‘T’ type frames were installed to support kiwifruit plants with a spacing of 3.00 m × 3.00 m. Female plants account for 8/9 of the total plants. The mean temperature, altitude and annual rainfall of the kiwifruit orchard was about 15–16 °C, 1267 m and 1293 mm, respectively. The loam soils (0–60 cm in deep) had 34.32 g kg^−1^ of organic matter, 1.41 g kg^−1^ of total nitrogen, 1.63 g kg^−1^ of total phosphorus, 1.13 g kg^−1^ of total potassium, 98.66 mg kg^−1^ of alkali–hydrolyzable nitrogen, 4.25 mg kg^−1^ of available phosphorus, 31.61 mg kg^−1^ of available iron, 18.96 mg kg^−1^ of available manganese, 48.78 mg kg^−1^ of total zinc, 18.02 cmol kg^−1^ of exchangeable calcium and 5.81 of pH value. 

#### 2.3.2. Field Control Experiment of Soft Rot Disease in Kiwifruit

The spray method was used to carry out the field control experiment of soft rot diseases in kiwifruit. The four experimental treatments: chitosan 100–time dilution liquid, 0.3% tetramycin AS 5000–time dilution liquid, chitosan 100 time + 0.3% tetramycin AS 5000–time dilution liquid and clear water (control). Clear water was derived from local irrigation water. A total of twelve plots were arranged randomly with three repetitions. Each plot contained eight trees and the interior six trees were used for measurements. According to our previous study, 20th May to 13th June and 2nd to 12th August were infection periods of soft rot pathogens on Guichang kiwifruit [12]. Thus, about 1.50 L and 2.00 L of fungicide dilution liquid was sprayed on kiwifruit plants (include fruit, bud, leaf and stem) at 19th May and 1st August, respectively. No rainfall was observed after 3–4 d of spray experiment. 

### 2.4. Determination of Control Effect, Development and Quality

The ripeness stage of Guichang kiwifruit is from about late September to middle October. A total of 200 kiwifruits from each plot were randomly collected on 1st October and divided into two groups, and stored at 25 ± 1 °C. One group was used for determining the incidence rate and control effect of soft rot in kiwifruit. Anther group was used to study the development, quality and aroma parameters of fruit. The incidence rate and control effect were respectively calculated using Equations (2) and (3).

Incidence rate (%) = 100 × Number of disease fruits/Total number of fruits(2)

Control effect (%) = 100 × (Incidence rate of control − Incidence rate of treatment)/Incidence rate of control(3)

### 2.5. Analytical Methods 

Total phenolic and total flavonoid contents, superoxide dismutase (SOD) and polyphenoloxidase (PPO) activities of fruits were determined as described by Zhang et al. [12]. Briefly, 2.00 g flesh sample was ground in 20 mL 1% (v/v) HCl–methyl alcohol at 3 °C and centrifuged (12,000× *g*, 10 min, 3 °C) when extracted for 1 h without light, and the contents of total phenolics and total flavonoids in the supernatant were determined at 280 nm and 325 nm, respectively; HCl–methyl alcohol is the standard. The development parameters such as longitudinal diameter, transverse diameter, lateral diameter, fruit shape index, single fruit volume and single fruit weight, as well as the quality parameters such vitamin C, total soluble sugar, soluble solid, dry matter, soluble protein and titratable acidity were also determined according to Zhang et al. [12]. 

The aroma components of fruits under the edible state were detected using gas chromatography–mass spectrometry (GC–MS, Trace 1300 GC, Thermo Fisher Scientific, Boston, MA, USA). Extract fiber head was 100 μm PDMS (Supelco, Bellefonte, PA, USA). Furthermore, 5 mL of fruit sample was put into in a 20 mL headspace bottle and the bottle cap was pressed tightly. After 40 min of headspace extraction at 55 °C, the sample was extracted by a solid–phase microextraction needle for 20 min. The sample was injected manually, and the extraction needle stayed at the injection port for 5 min. Chromatographic conditions: TG–5MS capillary column (30 m × 0.25 mm × 0.25 μm); helium (≥99.999%) was employed as carrier gas with a flow rate of 1.0 mL/min, no shunt injection; the column temperature was initiated at 50 °C (held for 2 min) and then increased to 240 °C at 4 °C/min (held for 5 min); sampling temperature was 240 °C. Mass spectrometry conditions: EI ionization source (70 eV), 1400 V multiplication voltage, 250 °C ion source temperature, 250 °C interface temperature and 40–450 m/z scanning range with interval 0.3 s.

### 2.6. Statistical Analyses

From each repetition, 100 kiwifruits were used for determining the incidence rate and control effect of soft rot in kiwifruit, and 100 kiwifruits were used for determining the development, quality and aroma parameters of fruits. All data were presented as means ± standard deviations (SD) of three repetitions. All analyses were carried on SPSS 18.0 (SPSS Inc., Chicago, IL, USA). A one–way analysis of variance (ANOVA) was used for determining the difference significances. Origin 10.0 was used to draw charts.

## 3. Results

### 3.1. Toxicity Effects of Chitosan and Tetramycin against Soft Rot Pathogens

The toxicity effects of tetramycin and chitosan on soft rot pathogens are shown in Table 1. Furthermore, 0.3% tetramycin AS possessed a superior antifungal activity for *B. dothidea* and *Phomopsis* sp. with EC_50_ values of 0.14 and 0.09 mg kg^−1^, while chitosan exhibited a relatively inferior toxicity potential for *B. dothidea* and *Phomopsis* sp. with EC_50_ values of 600.11 and 485.21 mg kg^−1^, respectively. 

### 3.2. Field Control Effect of Chitosan and Tetramycin on Soft Rot Disease of Kiwifruit

Table 2 depicts the field control effect of chitosan + tetramycin, tetramycin and chitosan on soft rot disease of kiwifruit. chitosan + tetramycin, tetramycin and chitosan significantly (*p* < 0.01) reduced the incidence rates of diseased fruit, chitosan + tetramycin was more effective. The control effect of soft rot disease by chitosan + tetramycin was 85.33%, which was higher than 80.99% for tetramycin and significantly (*p* < 0.01) higher than 40.66% for chitosan. chitosan + tetramycin and tetramycin exhibited a superior control effect for soft rot disease, while chitosan exhibited a relatively low control effect. These results indicate that chitosan could effectively enhance the control of soft rot by tetramycin, and that they had a significant additive effect. 

### 3.3. The Effects of Chitosan and Tetramycin on Total Phenolics, Total Flavonoids, SOD Activity and PPO Activity in Kiwifruit

**Figure 1:**
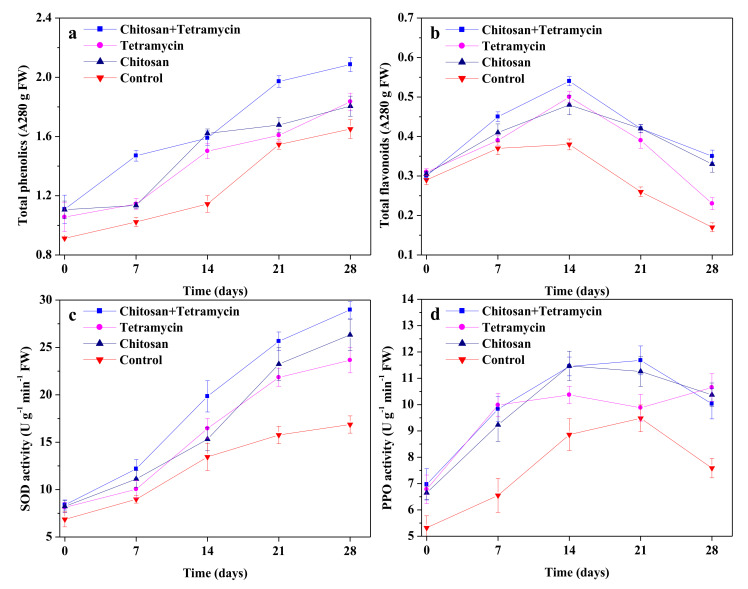
The effects of chitosan and tetramycin on the changes of total phenolics (**a**) and total flavonoids (**b**), SOD activity (**c**) and PPO activity (**d**) in kiwifruit during storage. Values and error bars indicate the mean and SD of three replicates, respectively.

The effects of chitosan + tetramycin, tetramycin and chitosan on total phenolics, total flavonoids, SOD activity and PPO activity in kiwifruit during storage are shown in Figure 1. chitosan + tetramycin, tetramycin and chitosan could effectively increase total phenolic and total flavonoid content of fruits, and enhance SOD and PPO activities of fruits. Total phenolics of fruits increased gradually with storage time, and total phenolics of fruits treated with chitosan + tetramycin were consistently significantly (*p* < 0.05) higher than those of tetramycin and control during storage. Total flavonoids of fruits increased firstly and then decreased during storage, and total flavonoids of fruits treated with chitosan + tetramycin were consistently significantly (*p* < 0.05) higher than those of tetramycin and control during storage (Figure 1b). Similarly, SOD activity of fruits treated with chitosan + tetramycin was consistently significantly (*p* < 0.05) higher than that of tetramycin, chitosan and control during storage (Figure 1c). The change trend of PPO activity in fruits treated with chitosan + tetramycin was similar to that of chitosan during storage, and higher than that of tetramycin during 7–21 days (Figure 1d). These findings emphasize that chitosan could significantly (*p* < 0.05) improve the promoting effects of tetramycin on total phenolics, total flavonoids and SOD activity in fruits. 

### 3.4. The Effects of Chitosan and Tetramycin on Growth and Quality of Kiwifruit

Table 3 displays the effects of chitosan and tetramycin on kiwifruit growth. Longitudinal diameter, transverse diameter, lateral diameter and fruit shape index of fruits were not significantly (*p* < 0.05) different in the four treatments. chitosan + tetramycin, tetramycin and chitosan effectively increased single fruit volume and significantly (*p* < 0.05) increased single fruit weight. The single fruit volume and weight of fruits treated with chitosan + tetramycin were 72.29 cm^3^ and 82.70 g, which effectively increased by 3.67, 2.55 or 7.18%, and 3.31, 3.27 or 7.70% compared to tetramycin, chitosan or control, respectively. These results indicate that the enhanced effects of kiwifruit growth with chitosan + tetramycin were higher than those of tetramycin and chitosan, and the combined application of chitosan and tetramycin significantly (*p* < 0.05) enhanced fruit growth and yield formation.

The effects of chitosan and tetramycin on kiwifruit quality are shown in Table 4. chitosan + tetramycin, tetramycin and chitosan significantly (*p* < 0.05) increased vitamin C, total soluble sugar, soluble solid, dry matter and soluble protein of fruits, as well as decreased titratable acidity of fruits compared to control. Vitamin C, total soluble sugar, soluble solid, dry matter and soluble protein of fruits treated with chitosan + tetramycin were higher than those of tetramycin and chitosan, however, there were no significant (*p* < 0.05) differences in the three treatments. The titratable acidity of fruits treated with chitosan + tetramycin was similar with that of chitosan, and lower than that of tetramycin. These findings indicate that chitosan + tetramycin could effectively improve kiwifruit quality, and chitosan and tetracycline had an additive effect on improving kiwifruit quality.

### 3.5. The Effects of Chitosan and Tetramycin on Aroma Compounds of Kiwifruit

The aromatic compounds of fruits were analyzed using GC–MS. As shown in Table 5, 60, 54, 56 and 49 aroma compounds in fruits treated with chitosan + tetramycin, tetramycin, chitosan and control were detected, respectively. The main aroma components in fruits treated with chitosan + tetramycin were butanoic acid ethyl ester, butanoic acid methyl ester, benzoic acid methyl ester, (E)–2–hexenal, benzoic acid ethyl ester, decamethyl–cyclopentasiloxane, hexanoic acid ethyl ester, nonanal and dodecamethyl–cyclohexasiloxane, etc. Additionally, the main aroma components in fruits treated with tetramycin were butanoic acid ethyl ester, butanoic acid methyl ester, benzoic acid methyl ester, benzoic acid ethyl ester, (E)–2–hexenal, decamethyl–cyclopentasiloxane, hexanoic acid ethyl ester and dodecamethyl–cyclohexasiloxane, etc. Additionally, the main aroma components in fruits treated with chitosan were butanoic acid ethyl ester, benzoic acid methyl ester, butanoic acid methyl ester, (E)–2–hexenal, benzoic acid ethyl ester, decamethyl–cyclopentasiloxane, hexanoic acid ethyl ester and dodecamethyl–cyclohexasiloxane, etc. While the main aroma components in fruits of control were butanoic acid ethyl ester, benzoic acid methyl ester, butanoic acid methyl ester, (E)–2–hexenal, decamethyl–cyclopentasiloxane, benzoic acid ethyl ester, octamethyl–cyclotetrasiloxane, nonanal and dodecamethyl–cyclohexasiloxane, etc. Butanoic acid ethyl ester, butanoic acid methyl ester, benzoic acid methyl ester, (E)–2–hexenal, benzoic acid ethyl ester, decamethyl–cyclopentasiloxane and dodecamethyl–cyclohexasiloxane were the common components with relatively high content in all treated fruits. Hexanoic acid ethyl ester was a relatively high specific component in fruits treated with chitosan + tetramycin, tetramycin and chitosan. These results indicate that chitosan + tetramycin could effectively improve aroma quality and increase aroma compounds of kiwifruit.

**Figure 2:**
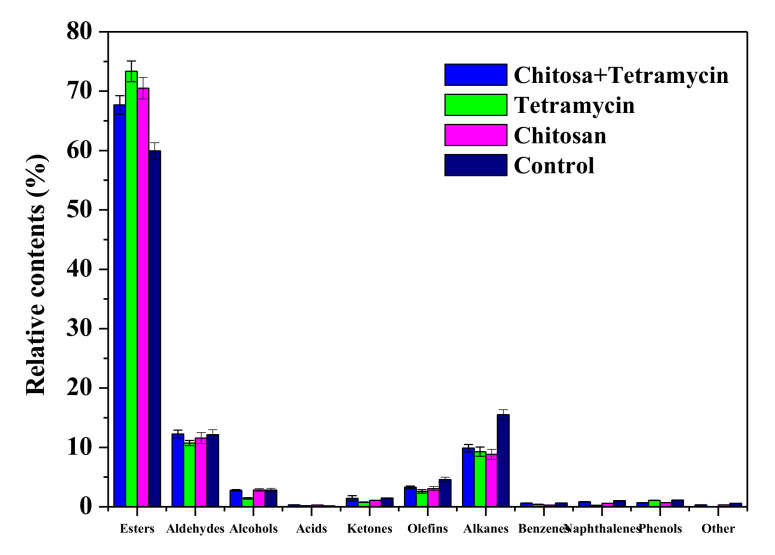
The effects of chitosan and tetramycin on the aromatic compound species of kiwifruit. Values and error bars indicate the mean and SD of three replicates, respectively.

The effects of chitosan and tetramycin on the aromatic compound species of kiwifruit are shown in Figure 2. The main aroma species in kiwifruit were esters, aldehydes, alkanes, olefins, alcohols and ketones. The relative contents of the esters, aldehydes, alkanes, olefins, alcohols and ketones in fruits treated with chitosan + tetramycin, tetramycin, chitosan and control were 67.69%, 73.35%, 70.51% and 59.94%; 12.26%, 10.74%, 11.56% and 12.14%; 9.87%, 9.28%, 8.82% and 15.52%; 3.27%, 2.57%, 3.05% and 4.58%; 2.77%, 1.42%, 2.84% and 2.81%; and 1.42%, 0.77%, 1.08% and 1.49%. chitosan + tetramycin, tetramycin and chitosan significantly (*p* < 0.05) increased the relative content of the esters and reduced the relative contents of the alkanes and olefins in fruits, tetramycin significantly (*p* < 0.05) reduced the relative contents of the aldehyde alcohols and ketones in fruits. These results emphasize that chitosan + tetramycin could effectively increase aroma diversity of kiwifruit. 

## 4. Discussion

Previous studies have shown that chitosan had antifungal activity on various fungal pathogens [28,34,35,36,37], and tetramycin could effectively inhibit the mycelial growth of *Botrytis cinerea*, *Colletotrichum scovillei*, *Pyricularia oryzae*, *Phytophthora capsici* and *Passalora fulva* [17,18,19,20,21]. The results here show that 0.3% tetramycin AS possessed a superior antifungal activity for *B. dothidea* and *Phomopsis* sp. with EC_50_ values of 0.14 and 0.09 mg kg^−1^, while chitosan exhibited a relatively inferior toxicity potential, respectively. The control effect of soft rot disease of kiwifruit with chitosan + tetramycin was 85.33%, which was higher than 80.99% of tetramycin and significantly (*p* < 0.01) higher than 40.66% of chitosan. Chitosan can trigger plant defense responses by inducing a variety of defense–related reactions [28,29,30,31,32,34,35,36,37]. Chitosan exhibited a limited control effect for soft rot disease of kiwifruit, which could be derived from the activation of kiwifruit defense response. Our previous results showed that 0.3% tetramycin AS possessed good field control efficacy for soft rot disease of kiwifruit [25]. In this study, chitosan could effectively enhance tetracycline against soft rot of kiwifruit, suggesting that chitosan and tetracycline had a significantly additive effect in the control of soft rot. Moreover, tetramycin, a mixture polyene antibiotic, unlikely results in resistance development [22,24]. Theoretically, the co–application of chitosan and tetramycin on soft rot in kiwifruit is more effective in preventing tetramycin resistance of pathogens.

Inducing disease resistance is an effective approach to control plant diseases, phenolics and flavonoids are important disease resistant substances in plants, SOD and PPO are also important defense enzymes associated with plant disease resistance [38]. Many studies reported that chitosan could increase total phenolics, total flavonoids and defense enzyme activity in dragon tomato, peach, pear, strawberry and jujube, which provide defense for plant cells to prevent pathogen infection [26,28,30,31,32,38]. Our previous results also show that chitosan could induce kiwifruit disease resistance [10,11,12,13]. Zhong et al. [39] reported that tetramycin could induce plant disease resistance by activating PPO, phenylalanine ammonia lyase (PAL) and peroxidase (POD) activities. Our previous results indicate that tetramycin increased total phenolics and total flavonoids, and activated SOD and PPO activities of kiwifruit [25]. The present results show that chitosan + tetramycin, tetramycin and chitosan could effectively increase total phenolics and total flavonoids content of fruits, and enhance SOD and PPO activities of fruits, which is consistent with the above studies. Moreover, total phenolics, total flavonoids and SOD activity of fruits treated with chitosan + tetramycin were consistently significantly (*p* < 0.05) higher than those of tetramycin during storage, and PPO activity of fruits treated with chitosan + tetramycin was higher than that of tetramycin during 7–21 days. These findings emphasize that chitosan could significantly improve the promoting effects of tetramycin on total phenolics, total flavonoids, SOD activity in fruits, as well as a significant additive effect of chitosan and tetracycline was available.

Chitosan can also be used as a nutrient to promote the growth of plants [29,33,34]. Our previous results show that chitosan enhanced the yield and quality of kiwifruit [10,11,12], tetramycin enhanced kiwifruit growth, improved kiwifruit quality and storability [25]. In this study, chitosan + tetramycin, tetramycin and chitosan significantly (*p* < 0.05) increased volume, weight, vitamin C, total soluble sugar, soluble solid, dry matter and soluble protein of kiwifruit, and decreased titratable acidity. The co–application of chitosan and tetramycin significantly (*p* < 0.05) enhanced fruit growth and yield formation, and exhibited an additive effect on improving kiwifruit quality. These beneficial effects may be closely related to their division of labor: tetramycin can protect kiwifruit from pathogen infection and promote the healthy growth of kiwifruit plants and fruits, chitosan can induce kiwifruit disease resistance and be used as a nutrient to promote kiwifruit growth.

Aroma compounds are an important part of kiwifruit quality, and an important factor for affecting the fresh eating, storage and processing of kiwifruit [40,41]. More than 90 aroma compounds have been reported in kiwifruit, among which butyrate ethyl, (E)–2–hexenal and hexanal are considered to be the three most important volatile components that determine kiwifruit aroma [42,43]. Our previous results indicate that the main aroma compounds in ‘Guichang’ kiwifruit were butanoic acid ethyl ester, butanoic acid methyl ester, benzoic acid methyl ester, (E)–2–hexenal, benzoic acid ethyl ester, etc. [44]. In this study, 60, 54, 56 and 49 aroma compounds in fruits treated with chitosan + tetramycin, tetramycin, chitosan and control were detected, respectively. Butanoic acid ethyl ester, butanoic acid methyl ester, benzoic acid methyl ester, (E)–2–hexenal and benzoic acid ethyl ester are the main aroma compounds in fruits, which is consistent with our previous report. The high contents of butanoic acid ethyl ester, butanoic acid methyl ester and benzoic acid methyl ester may be the main factors that distinguish ‘Guichang’ kiwifruit from other kiwifruits, which endows it with a unique and rich banana–pineapple fragrance. Moreover, hexanoic acid ethyl ester was a relatively high specific component in fruits treated with chitosan + tetramycin, tetramycin and chitosan. Simultaneously, chitosan + tetramycin, tetramycin and chitosan significant (*p* < 0.05) increased the relative content of the esters and reduced the relative contents of the alkanes and olefins in fruits, tetramycin significantly (*p* < 0.05) reduced the relative contents of the aldehyde alcohols and ketones in fruits. These results indicate that chitosan + tetramycin could effectively improve aroma quality and increase aroma compounds and diversity of kiwifruit.

Chitosan is a natural macromolecule compound with antibacterial, nontoxic, antioxidant, renewable and biocompatible advantages, and tetramycin is a novel, eco–friendly and low–toxicity agriculture antibiotic. Moreover, the concentration of tetramycin used in the field experiment was very low (0.3% tetramycin AS 5000–time dilution liquid), and the safe interval (1st August to 1st October, 60 d) and soft ripening (more than 20 d) periods of kiwifruit were very long. Therefore, the food safety risks caused by tetramycin or chitosan are almost nonexistent. This study highlights that the co–application of chitosan and tetramycin is an effective, green, safe and economical approach for avoiding pathogen resistance, controlling kiwifruit soft rot, improving kiwifruit growth and quality and increasing kiwifruit aroma. 

## 5. Conclusions

In conclusion, chitosan could effectively enhance tetramycin against soft rot of kiwifruit, and improve the promoting effects of tetramycin on total phenolics, total flavonoids and SOD activity in fruits. Moreover, the co–application of chitosan and tetramycin was more effective than tetramycin or chitosan alone in enhancing fruit growth, improving fruit quality and increasing fruit aroma. This work highlights that chitosan can be used as an adjuvant to enhance tetramycin against soft rot of kiwifruit and promote tetramycin’s improvement for kiwifruit growth, quality and aroma.

## Figures and Tables

**Table 1:** Toxicities of tetramycin and chitosan against *B. dothidea* and *Phomopsis sp*.

Pathogens	Antibiotic Bactericides	Regression Equation	Determination Coefficient (*R*^2^)	EC_50_ (mg kg^−1^)
Chitosan > *B. dothidea*	0.3% Tetramycin AS	*y* = 6.0759 + 1.2511 *x*	0.9963	0.14
	Chitosan	*y* = 5.1568 + 0.7069 *x*	0.9941	600.11
*Phomopsis* sp.	0.3% Tetramycin AS	*y* = 1.1510 + 9.3601 *x*	0.9968	0.09
	Chitosan	*y* = 5.2904 + 0.9250 *x*	0.9865	485.41

*x* and *y* indicate the tetramycin (or chitosan) concentration and inhibition rate, respectively.

**Table 2:** The control effects of tetramycin and chitosan on soft rot diseases of kiwifruit.

Treatments	Incidence Rate of Disease Fruit (%)	Control Effect (%)
Chitosan + tetramycin	8.00 ± 2.00 ^cC^	85.33 ± 2.12 ^aA^
Tetramycin	10.00 ± 2.65 ^cC^	80.99 ± 6.86 ^aA^
Chitosan	32.00 ± 3.61 ^bB^	40.66 ± 3.29 ^bB^
Control	58.00 ± 3.61 ^aA^	

Values indicate the mean ± SD of three replicates. Different small letters indicate significant differences at 5% level (*p* < 0.05), and different capital letters indicate significant differences at 1% level (*p* < 0.01).

**Table 3:** The effects of chitosan and tetramycin on the development of kiwifruit.

Treatments	Longitudinal Diameter (mm)	Transverse Diameter (mm)	Lateral Diameter (mm)	Fruit Shape Index	Single Fruit Volume (cm^3^)	Single Fruit Weight (g)
Chitosan + Tetramycin	76.93 ± 0.47 ^a^	52.97 ± 1.42 ^a^	42.35 ± 0.72 ^a^	1.61 ± 0.03 ^a^	72.29 ± 3.52 ^a^	82.70 ± 0.56 ^a^
Tetramycin	76.12 ± 0.14 ^a^	52.02 ± 0.61 ^a^	42.06 ± 0.24 ^a^	1.62 ± 0.02 ^a^	69.73 ± 1.08 ^ab^	80.05 ± 0.82 ^b^
Chitosan	76.47 ± 0.22 ^a^	52.27 ± 0.53 ^a^	42.13 ± 0.81 ^a^	1.62 ± 0.00 ^a^	70.49 ± 1.12 ^ab^	80.08 ± 0.74 ^b^
Control	75.51 ± 0.33 ^ab^	51.71 ± 0.50 ^a^	41.26 ± 0.36 ^a^	1.62 ± 0.02 ^a^	67.45 ± 1.17 ^b^	76.79 ± 1.59 ^c^

Values indicate the mean ± SD of three replicates. Small letters indicate significant differences at 5% level (*p* < 0.05).

**Table 4:** The effects of chitosan and tetramycin on quality of kiwifruit.

Treatments	Vitamin C (g kg^−1^)	Total Soluble Sugar (%)	Soluble Solid (%)	Dry Matter (%)	Soluble Protein (%)	Titratable Acidity (%)
Chitosan + tetramycin	1.91 ± 0.02 ^a^	12.61 ± 0.24 ^a^	15.60 ± 0.10 ^a^	19.56 ± 0.09 ^a^	1.78 ± 0.04 ^a^	1.04 ± 0.03 ^b^
Tetramycin	1.88 ± 0.01 ^a^	12.40 ± 0.07 ^a^	15.20 ± 0.10 ^a^	19.14 ± 0.10 ^ab^	1.75 ± 0.02 ^a^	1.09 ± 0.02 ^b^
Chitosan	1.89 ± 0.01 ^a^	12.56 ± 0.11 ^a^	15.40 ± 0.10 ^a^	19.37 ± 0.16 ^a^	1.77 ± 0.02 ^a^	1.05 ± 0.01 ^b^
Control	1.81 ± 0.01 ^b^	12.08 ± 0.01 ^b^	14.37 ± 0.06 ^b^	18.34 ± 0.03 ^c^	1.72 ± 0.03 ^b^	1.16 ± 0.04 ^a^

Values indicate the mean ± SD of three replicates. Small letters indicate significant differences at 5% level (*p* < 0.05).

**Table 5:** The effects of chitosan and tetramycin on the aromatic compounds of kiwifruit.

Aroma Components	Relative Contents/%			
	Chitosan + Tetramycin	Tetramycin	Chitosan	Control
Ethyl acetate	0.30 ± 0.01	0.29 ± 0.01	0.34 ± 0.01	–
Butanoic acid methyl ester	16.07 ± 1.08	15.7 ± 1.03	16.11 ± 1.21	14.52 ± 1.75
Butanoic acid ethyl ester	22.09 ± 1.21	21.7 ± 1.25	22.21 ± 1.35	20.41 ± 1.88
Hexanoic acid methyl ester	1.35 ± 0.01	1.31 ± 0.01	1.04 ± 0.02	1.78 ± 0.12
Hexanoic acid ethyl ester	3.06 ± 0.13	4.62 ± 0.42	2.88 ± 0.13	–
Butanoic acid 2–methyl propyl ester	0.51 ± 0.01	0.44 ± 0.01	0.47 ± 0.02	–
2–Furancarboxylic acid ethyl ester	0.33 ± 0.01	0.32 ± 0.02	0.33 ± 0.01	0.28 ± 0.05
Benzoic acid methyl ester	15.64 ± 1.03	15.54 ± 1.39	16.77 ± 1.25	15.36 ± 1.55
Octanoic acid methyl ester	1.13 ± 0.04	1.20 ± 0.01	1.22 ± 0.03	1.10 ± 0.68
Benzoic acid ethyl ester	6.03 ± 0.87	10.97 ± 0.33	7.88 ± 0.45	5.40 ± 1.01
Octanoic acid ethyl ester	0.75 ± 0.04	0.35 ± 0.03	0.67 ± 0.32	0.72 ± 0.35
Benzoic acid hexyl ester	–	0.05 ± 0.00	–	–
Butyl benzoate	0.18 ± 0.11	0.69 ± 0.04	0.33 ± 0.23	0.18 ± 0.11
Decanoic acid ethyl ester	0.03 ± 0.00	0.03 ± 0.00	0.03 ± 0.00	–
Octadecyl 2–amyl sulfate	0.03 ± 0.00	0.03 ± 0.00	0.02 ± 0.00	–
2– methyl propyl phthalate	0.03 ± 0.00	0.03 ± 0.00	0.03 ± 0.00	–
Dibutyl phthalate	0.11 ± 0.01	0.04 ± 0.00	0.15 ± 0.01	0.19 ± 0.04
2–Butenedioic acid (E)–, bis(2–ethylhexyl) ester	0.05 ± 0.01	0.04 ± 0.00	0.03 ± 0.00	–
(E) –2–hexenal	9.07 ± 0.34	8.78 ± 0.20	8.94 ± 0.26	7.97 ± 1.20
Benzeneacetaldehyde	–	0.08 ± 0.01	0.07 ± 0.00	–
Nonanal	2.55 ± 0.85	1.52 ± 0.05	1.88 ± 0.07	3.55 ± 0.83
Decanal	0.62 ± 0.11	0.28 ± 0.03	0.58 ± 0.04	0.62 ± 0.11
2–Undecenal	–	0.05 ± 0.00	0.07 ± 0.00	–
Fifteen aldehydes	0.02 ± 0.00	0.03 ± 0.00	0.02 ± 0.00	–
1–Deoxy–d–arabitol	0.81 ± 0.04	–	0.80 ± 0.02	0.77 ± 0.11
Eucalyptol	1.13 ± 0.22	1.05 ± 0.07	1.19 ± 0.06	1.22 ± 0.24
à–Terpineol	0.31 ± 0.01	0.29 ± 0.01	0.27 ± 0.01	0.23 ± 0.01
(E)–4, 6–dimethyl–1–methyl sulfide –1, 5–heptadiene –4–alcohol	–	0.08 ± 0.01	–	–
Trans –2, 6–dimethyl–6 – (p–methyl–phenyl) –heptenol	0.11 ± 0.01	–	0.13 ± 0.01	0.13 ± 0.02
(Z)–13– docosahlenol	0.41 ± 0.01	–	0.45 ± 0.01	0.46 ± 0.05
E–2–Hexenyl benzoate	0.16 ± 0.02	–	0.17 ± 0.02	0.15 ± 0.04
Hexanoic acid anhydride	–	0.04 ± 0.00	–	–
2– methyl pentahydride	0.15 ± 0.01	0.12 ± 0.01	0.12 ± 0.01	–
7– isopropyl – dicyclic [0,3,3] octane–2–ketone	0.21 ± 0.01	–	–	0.21 ± 0.01
3–tert–Butyl–2–pyrazolin–5–one	–	0.04 ± 0.00	–	–
(S)– 6– (1– methyl vinyl)– cyclohexenone	0.13 ± 0.01	–	0.15 ± 0.01	0.11 ± 0.02
Beta–Malaysia	0.10 ± 0.01	0.06 ± 0.01	0.05 ± 0.01	0.11 ± 0.01
Trans geranyl acetone	0.31 ± 0.02	0.29 ± 0.03	0.34 ± 0.01	0.33 ± 0.02
trans–á–Ionone	0.15 ± 0.01	0.06 ± 0.01	0.16 ± 0.01	0.17 ± 0.02
3–(2–pentene)–1,2,4–cyclopentaerone	0.52 ± 0.01	0.24 ± 0.03	0.38 ± 0.01	0.56 ± 0.03
Benzophenone	–	0.08 ± 0.01	–	–
1,7, 7–trimethyl–hept–2–ene	0.38 ± 0.02	0.27 ± 0.02	0.33 ± 0.02	1.27 ± 0.13
à–Cubebene	0.98 ± 0.05	1.15 ± 0.05	1.23 ± 0.02	0.76 ± 0.11
(E)–á–Famesene	0.17 ± 0.04	–	–	0.16 ± 0.04
isoledene	0.18 ± 0.03	–	0.18 ± 0.03	0.15 ± 0.02
cis–Calamenene	1.07 ± 0.14	1.12 ± 0.01	1.16 ± 0.01	1.66 ± 0.14
à–Calacorene	0.32 ± 0.02	–	–	0.46 ± 0.07
isoaromadendrene epoxide	0.14 ± 0.01	–	0.12 ± 0.01	0.12 ± 0.02
Neophytadiene	0.03 ± 0.00	0.03 ± 0.00	0.03 ± 0.00	–
Octamethyl– cyclotetrasiloxane	1.48 ± 0.06	0.04 ± 0.00	0.03 ± 0.00	5.15 ± 1.53
Decamethyl– cyclopentasiloxane	4.52 ± 0.18	5.27 ± 0.64	4.89 ± 0.64	6.08 ± 1.30
1,1–Dichloro–2–methyl–3–(4,4–diformyl–1,3–butadien–1–yl) cyclopropane	–	0.03 ± 0.00	–	–
Dodecamethyl– cyclohexasiloxane	2.05 ± 0.12	2.46 ± 0.19	2.33 ± 0.13	2.55 ± 0.33
Tetradecane	0.22 ± 0.01	–	0.25 ± 0.01	0.27 ± 0.03
Tetradecamethyl– cycloheptasiloxane	1.23 ± 0.13	1.00 ± 0.18	1.08 ± 0.11	1.06 ± 0.13
Hexadecamethyl– cyclooctasiloxane	0.21 ± 0.01	0.32 ± 0.04	0.12 ± 0.01	0.29 ± 0.01
Octadecamethyl– cyclononasiloxane	0.16 ± 0.01	0.12 ± 0.02	0.12 ± 0.02	0.12 ± 0.01
Eicosamethyl– cyclodecasiloxane	–	0.04 ± 0.01	–	–
1, 4–bis – (1–methylethyl) benzene	0.13 ± 0.01	–	0.04 ± 0.01	0.11 ± 0.01
1, 1–propane diphenyl	–	0.04 ± 0.01	–	–
1, 1–2–butene –1, 4–2–benzene	0.21 ± 0.01	0.26 ± 0.06	0.22 ± 0.02	0.23 ± 0.02
2 – methyl naphthalene	0.04 ± 0.00	0.04 ± 0.01	0.03 ± 0.01	–
1, 7–dimethyl naphthalene	0.22 ± 0.01	0.05 ± 0.01	0.28 ± 0.01	0.29 ± 0.02
1–Isopropyl–4,7–dimethyl–1,2,3,4,5,6–hexahydronaphthalene	0.29 ± 0.03	–	–	0.28 ± 0.04
1,6–dimethyl–4 – (1–methylethyl) naphthalene	0.37 ± 0.03	0.24 ± 0.09	0.27 ± 0.01	0.57 ± 0.09
1,2,3,4–tetramethene	0.17 ± 0.02	–	–	0.18 ± 0.01
Butylated Hydroxytoluene	0.38 ± 0.02	0.05 ± 0.01	0.44 ± 0.01	0.32 ± 0.02
(S)– (S) –2–methyl–5 – (1,2, 2–tricyclopentyl) phenol	0.29 ± 0.10	1.03 ± 0.04	0.25 ± 0.01	0.81 ± 0.10
Dibenzyl ketoxime	0.31 ± 0.01	–	0.32 ± 0.01	0.34 ± 0.03
(Z) –oleate amide	–	–	–	0.24 ± 0.01

Values indicate the mean of three replicates.

## Data Availability

The datasets during or analyzed during the current study available from the corresponding author on reasonable request.
